# Suture Granuloma Showing False-Positive Findings on FDG-PET

**DOI:** 10.1155/2013/472642

**Published:** 2013-05-22

**Authors:** Kohei Takahara, Hiroaki Kakinoki, Saya Ikoma, Kazuma Udo, Shohei Tobu, Yuji Satoh, Yuji Tokuda, Mitsuru Noguchi, Shigehisa Aoki, Jiro Uozumi

**Affiliations:** ^1^Department of Urology, Faculty of Medicine, Saga University, Nabeshima 5-1-1, Saga 849-8501, Japan; ^2^Department of Pathology and Microbiology, Saga University, Nabeshima 5-1-1, Saga 849-8501, Japan

## Abstract

We report a case of a 33-year-old male with a mixed germ-cell testicular tumor. Postoperative follow-up FDG-PET revealed concentration of FDG in the left inguinal area which is not tumor metastasis or local recurrence but suture reactivity granuloma. In this paper, we reviewed suture granulomas associated with false-positive findings on FDG-PET after surgery. If FDG-PET will be used more frequently in the future, it will be necessary to refrain from using silk thread in order to prevent any unnecessary surgery.

## 1. Introduction

Imaging studies are fundamental to the diagnosis and planning of therapy for testicular tumors. FDG-PET (18F-fluorodeoxy glucose-positron emission tomography) is indicated extensively and serves a very useful purpose in detecting malignant tumors and evaluating therapy. 

However, the use of FDG-PET based on glucose metabolism understandingly produces false-positive results in cases with inflammatory or granulation tissue and can occasionally confuse clinicians.

This report presents the case of suture reactivity granuloma showing a false-positive finding on postoperative follow-up FDG-PET after high orchiectomy in a patient with a mixed germ-cell tumor that complicated both the diagnosis of recurrent testicular tumors and the selection of the optimal therapeutic alternatives.

## 2. Case Report

A 33-year-old male was referred to the urological department for treatment of ongoing edema in both legs and left testicular enlargement. A physical examination revealed the left testis to be enlarged to the size of a fist with a hard, irregular surface and left subclavian lymph node swelling. A complete blood count was within the normal limits. The C-reactive protein level was increased to 3.84 mg/dL. The serum human chorionic gonadotropin (hCG) level was significantly elevated to 3,360 mIU/mL and the *β*-hCG level was 19.5 ng/mL. The alpha fetoprotein level was 3 ng/mL, overlapping the normal range. The LDH level was increased to 1,690 IU/L. Abdominal-pelvic CT performed on the initial visit revealed a left testicular tumor with left inguinal and para-aortic lymph node swelling. The patient was diagnosed with a stage IIIc testicular tumor and immediately underwent high orchiectomy. The histopathological diagnosis was a mixed germ-cell tumor (seminoma and embryonal carcinoma). 

**Figure 1:**
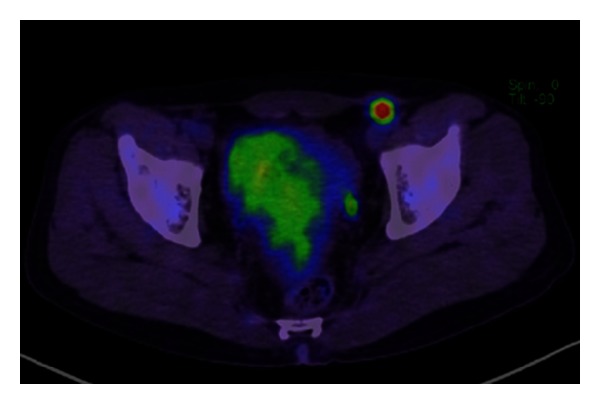
FDG-PET revealed concentration of FDG in the left inguinal area. The size of the mass was 1.1 cm.

**Figure 2:**
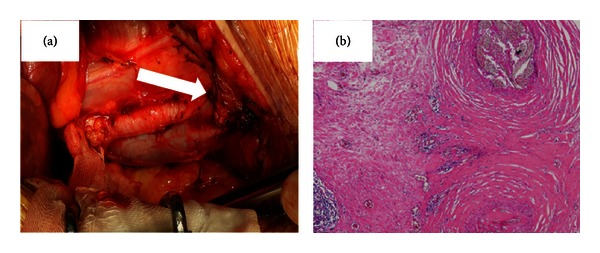
(a) The intraoperative findings revealed tumors near the left femoral artery and vein (arrow). (b) The histopathological findings revealed granulation tissue around the silk ligature surrounding lymphocytic infiltration ×100.

The patient received three courses of BEP (Bleomycin, Etoposide, and Cisplatin) therapy, and one course of EP (Etoposide and Cisplatin) therapy was added as postoperative chemotherapy. The levels of tumor markers returned to the normal ranges and became negative one month later, and abdominal-pelvic CT revealed significant reductions of the lymph nodes and liver metastases. Four courses of VIP (Etoposide, Ifomide, and Cisplatin) therapy were administered as additional chemotherapy for residual para-aortic lymph node swelling. Although follow-up CT revealed no significant enlargement of lymph nodes or the presence of metastases, FDG-PET revealed a concentration of FDG in the left inguinal area, which was a surgical site (Figure 1). Therefore, the area was suspected to be a site of tumor metastasis or local recurrence. Left inguinal lymph node dissection was performed (Figure 2(a)), and the histopathological findings revealed suture granuloma without either any viable cells or testicular tumor recurrence (Figure 2(b)).

## 3. Discussion

Although FDG-PET was originally thought to be less useful for detecting urological malignancies because FDG is eliminated via the kidneys, some reports have demonstrated the usefulness of FDG-PET for detecting urological malignancies. Hinz et al. suggested that FDG-PET is useful under specific conditions, such as when the size of the residual mass is greater than 3 cm; however, because the possibility of false-positives is not low, it is necessary to be careful when determining the indications for surgical treatment of residual masses [1]. De Santis and Pont also reported that it is difficult to distinguish necrotic tissue from a mature teratoma and that the usefulness of FDG-PET in detecting testicular tumors after surgery is limited [2]. Another report stated that FDG-PET has a relatively high sensitivity (80%), specificity (100%), positive predictive rate (100%) and negative predictive rate (95%) in the postoperative followup of patients with seminoma, making the modality very useful [3]. However, FDG-PET has no clinical benefits compared with CT or the tumor marker levels regarding the postoperative followup of nonseminoma cases [4]. 

The treatment guidelines for testicular tumors issued by the European Association of Urology and The National Comprehensive Cancer Network do not recognize the usefulness of FDG-PET for staging or the postoperative followup of nonseminoma disease. This is because the use of FDG-PET can lead to the unnecessary treatment of patients due to the accumulation of inflammatory and granulation tissue. In fact, some reports of suture granulomas associated with false-positive findings on FDG-PET after surgery have been published (Table 1). All of these reports revealed that positive findings on FDG-PET can be confused with tumor metastasis. It is therefore necessary to consider the possibility of suture reactive granulomas in cases of FDG-positive findings at the surgical site in patients with nonseminoma tumors.

Chung et al. reported a case in which they performed an aspiration biopsy using ultrasound and diagnosed FDG-PET-positive findings as a suture granuloma without performing open surgery, while, in another case, they diagnosed a suture granuloma using open surgery [5–8]. In cases of testicular tumors, suture reactive granulomas can originate from the inguinal area because high orchiectomy is performed as the first treatment in most cases. Therefore, if FDG-positive findings originate from the inguinal area after high orchiectomy, aspiration biopsies using ultrasound can be useful in diagnosing suture reactive granulomas without the use of open surgery because it is easier to perform an aspiration biopsy of the inguinal area than other sites. However, in cases where FDG-positive findings are recognized around femoral vessels and within the intraperitoneal area, open surgery must be performed to confirm whether these findings indicate tumor recurrence. Therefore, if FDG-PET will be used more frequently in the future, it will be necessary to refrain from using silk thread in order to prevent any unnecessary surgery. 

Some studies have reported that absorption thread, including manufactured silk, can be a more frequent cause of suture granulomas than nonabsorption thread [9, 10]. Moreover, Iwase et al. demonstrated that suture granulomas that develop following absorption thread suturing heal following simple percutaneous drainage within one week, whereas suture granulomas that develop following braided silk suturing require an average of 16 days to heal and necessitate the removal of the infected suture strings in all cases [9]. Absorption thread suturing rarely causes suture granulomas, and this type granuloma can be diagnosed and treated with simple drainage. Therefore, using absorption thread is beneficial for preventing unnecessary surgery.

 In conclusion, PET is useful for the postoperative followup of seminoma; however, sufficient evidence has not yet been accumulated for nonseminoma cases. Recently, other case reports similar to ours of suture granulomas in other organs have been published. Therefore, clinicians must consider the possibility for suture granulomas in cases with positive findings on PET detected within the surgical site during postoperative followup of nonseminoma. Of course, tumor markers must be evaluated in order to judge whether postoperative positive findings on PET indicate suture granulomas or tumor recurrence. However, clinicians have no choice but to perform tissue diagnosis at present. Therefore, large-scale studies of the effectiveness and impact of PET in the postoperative followup of testicular tumors are needed in the future. 

## Figures and Tables

**Table 1:** Previous case reports of suture granulomas showing false-positive findings on FDG-PET after surgery.

No.	Year	Author	Age, gender	Primary disease	Treatment	Interval^a^	Pathologic/clinical result	Tumor marker
1	2005	Lim et al. [6]	61, F	Breast cancer	Surgical resection	24 months	Suture granuloma by silk	Positive
2	2006	Chung et al. [5]	39, F	Thyroid cancer	Ultrasound guided aspiration biopsy	6 months	Suture granuloma	Not mentioned
3	2007	Yüksel et al. [8]	42, M	Pneumothorax	Surgical resection	15 years	Suture granuloma by nonabsorbable material	Not performed
4			47, M	Lung cancer	Surgical resection	8 months	Suture granuloma	Not mentioned
5	2012	Kikuchi et al. [7]	64, F	Hypopharyngeal cancer	Excisional biopsy	35 months	Suture granuloma by silk	Negative
6			71, M	Oropharyngeal cancer	Excisional biopsy	38 months	Suture granuloma by silk	Negative

^a^The time interval between the primary operation and the radiological diagnosis of the lesion.
